# The Detection of Periodic Reemergence Events of SARS-CoV-2 Delta Strain in Communities Dominated by Omicron

**DOI:** 10.3390/pathogens11111249

**Published:** 2022-10-28

**Authors:** Claire E. Westcott, Kevin J. Sokoloski, Eric C. Rouchka, Julia H. Chariker, Rochelle H. Holm, Ray A. Yeager, Joseph B. Moore, Erin M. Elliott, Daymond Talley, Aruni Bhatnagar, Ted Smith

**Affiliations:** 1 Department of Microbiology and Immunology, School of Medicine, University of Louisville, 505 S. Hancock St., Louisville, KY 40202, USA; 2 Center for Predictive Medicine for Biodefense and Emerging Infectious Disease (CPM), University of Louisville, 505 S. Hancock St., Louisville, KY 40202, USA; 3 Department of Biochemistry and Molecular Genetics, School of Medicine, University of Louisville, 323 E. Chestnut St., Louisville, KY 40202, USA; 4 KY INBRE Bioinformatics Core, University of Louisville, 522 E. Gray St., Louisville, KY 40202, USA; 5 Christina Lee Brown Envirome Institute, University of Louisville, 302 E. Muhammad Ali Blvd., Louisville, KY 40202, USA; 6 Diabetes and Obesity Center, School of Medicine, University of Louisville, 580 S. Preston St., Louisville, KY 40202, USA; 7 Louisville/Jefferson County Metropolitan Sewer District, Morris Forman Water Quality Treatment Center, 4522 Algonquin Parkway, Louisville, KY 40211, USA

**Keywords:** next-generation sequencing, public health, SARS-CoV-2, variant detection, wastewater

## Abstract

Despite entering an endemic phase, SARS-CoV-2 remains a significant burden to public health across the global community. Wastewater sampling has consistently proven utility to understanding SARS-CoV-2 prevalence trends and genetic variation as it represents a less biased assessment of the corresponding communities. Here, we report that ongoing monitoring of SARS-CoV-2 genetic variation in samples obtained from the wastewatersheds of the city of Louisville in Jefferson county Kentucky has revealed the periodic reemergence of the Delta strain in the presence of the presumed dominant Omicron strain. Unlike previous SARS-CoV-2 waves/emergence events, the Delta reemergence events were geographically restricted in the community and failed to spread into other areas as determined by wastewater analyses. Moreover, the reemergence of the Delta strain did not correlate with vaccination rates as communities with lower relative vaccination have been, to date, not affected. Importantly, Delta reemergence events correlate with increased public health burdens, as indicated by increased daily case rates and mortality relative to non-Delta wastewatershed communities. While the underlying reasons for the reemergence of the Delta variant remain unclear, these data reaffirm the ongoing importance of wastewater genomic analyses towards understanding SARS-CoV-2 as it enters the endemic phase.

## 1. Introduction

Throughout the COVID-19 Pandemic, many developed and developing communities have relied on surveillance programs to monitor the prevalence/burden of SARS-CoV-2 infection and to define the genetic composition of the circulating SARS-CoV-2 strains [1,2,3,4]. These efforts have included the assessments of clinical samples as well as the unbiased surveillance of wastewater samples to determine infection dynamics over specified geographic scales [5]. Using a dual approach to identify variants in the community by testing samples obtained from patients or from smaller community wastewater catchments allows for an improved public health response and a better understanding of the relationships between subpopulation wastewater samples and community-wide infection incidence [6]. Indeed, there have been several notable instances in the local community and communities at large where “wastewater screening and clinical confirmation” has proven to be of tremendous benefit towards identifying and implementing effective public health responses to mitigate the impact of SARS-CoV-2 in the community [7]. 

As the pandemic has begun to enter the endemic phase in many global communities, clinical and public health efforts have largely focused on the Omicron variant. This focus is understandable since the Omicron variant and its associated sublineages have become the dominant strain of SARS-CoV-2 [8]. The explosive emergence of the Omicron variant, likely due to the increased rate of transmission associated with the variant and the reduced efficacy of the vaccines available at the time against Omicron infection, resulted in significant surges in developed and developing communities. The magnitude of this emergence event has led many to postulate that significant herd immunity may exist in populations affected by the Omicron surge. Nonetheless, several studies have indicated that reciprocal immunity between adaptive immune responses spurred by infections of the Omicron variant may not effectively limit infections of other SARS-CoV-2 variants, and vice versa [9,10,11,12]. Thus, it remains important that public health monitoring continue to include unbiased assessments of the SARS-CoV-2 variants within a population. 

Here, we report the findings of ongoing SARS-CoV-2 wastewater monitoring efforts of a robust partnership between the University of Louisville’s Envirome Institute, the Rockefeller Foundation’s Pandemic Prevention Institute, and the city of Louisville KY. Importantly, these efforts have led to the detection of a series of reemergence events in the city of Louisville KY after the dominant emergence of the Omicron variant, Altogether, these observations indicate that it is important to continue the observation of SARS-CoV-2 community infection by wastewater surveillance and underscore the im-portance of wastewater monitoring to pandemic readiness and response.

## 2. Materials and Methods

### 2.1. Wastewater Sampling and Preparation

Aggregate samples from the five major water quality treatment centers servicing the city of Louisville in Jefferson County Kentucky were collected using a composite sampler, with sample volumes collected over a 24-h period with a rate of sampling was 30 mL per 15-min period. The composite samples were then homogenized and aliquoted prior to further assessment. Samples were maintained on ice and processed within 12 h of collection. Each 45 mL sample was initially passed through a 70-micron filter (VWR; 76327-100) and PEG-8000 (Millipore-Sigma 89510, Burlington, MA, USA) and NaCl were added to a final concentration of 12.5 and 210 mM, respectively. The samples were thoroughly mixed and refrigerated overnight prior to being subjected to centrifugation at 15,000× *g* for 30 min at 4 °C. The supernatant was discarded, and the pellet was resuspended in 1.1 mL of TRIzol (Thermo Scientific, Waltham, MA, USA; #15596018) and transferred to a sterile microcentrifuge tube. After vortexing to ensure complete resuspension, the sample was incubated for 5 min at room temperature and then clarified via centrifugation at 12,000× *g* for 5 min at 4 °C. The sample was then divided into two new microcentrifuge tubes, with one half of the sample processed further, and the other half archived at −80 °C for later assessment.

A total of 500 uL of TRIzol was added to the sample being processed, and the sample was processed using a Direct-zol 96 MagBead RNA kit (Zymo Research, Irvine, CA, USA; R2102) according to the manufacturer’s instructions. The RNA sample was eluted into 50 uL of molecular grade water, and RNA cleanup was achieved using the Zymo RNA Clean & Concentrator-5 kit (Zymo Research; R1016) according to the manufacturer’s instructions. The final sample yield was 62 uL of molecular grade water, and the purity/concentration of the extracted RNA was determined using a NanoDrop 1000. The sample was then divided into aliquots for two purposes- the quantification of viral load via qRT-PCR, and the development of cDNA libraries for next-generation sequencing. 

### 2.2. cDNA and Library Synthesis

From the above samples, cDNA was synthesized using the Superscript IV First-Strand Synthesis System (Thermo Fisher; #18091050) using random hexamer priming. The RT reactions were assembled as according to the manufacturer’s instructions, with the final RT reaction being 40 uL in volume, 7.5 uL of which was the purified RNA sample from above. The reverse transcription reaction cycling conditions were slightly modified, as recommended by the Swift Biosciences SNAP low input protocol (Swift Bioscience, Ann Arbor, MI, USA; COSG1 V2-96 SN-5 × 296). Briefly, sequential incubations of 23 °C for 10 min, 50 °C for 30 min, and 80 °C for 10 min were used. 

Libraries were prepared using the Swift Biosciences SNAP low input protocol for SARS-CoV-2 to generate whole genome amplicons. Briefly, 10 uL of cDNA was combined with 20 uL of reaction mix to proceed with the multiplex PCR according to the manufacturer’s instructions. To increase yield, one additional amplification cycle was performed. The resulting PCR amplicons were size selected using SPRIselect beads (Beckman Coulter, Brea, CA, USA; B23318) at a 1:1 ratio. Purified amplicons were resuspended in 17.4 uL of RNAse-free TE buffer. Individual samples were indexed via PCR using the SNAP Unique Dual Indexing Primers (Swift Bioscience; SN91096-1-PLATE). Again, to increase yield, two additional amplification cycles were performed. The resulting crude libraries were further cleaned using a 0.65 × PEG NaCl purification. The purified libraries were then eluted in 22 uL of TE buffer and stored at −20 °C until needed. 

Libraries were normalized using Swift Biosciences Normalase 2 nM final pooling protocol, as per the manufacturer’s instructions.

### 2.3. Next-Generation Sequencing and Data Analysis

Library pools were spike with PhiX were denatured and diluted as per Illumina’s directions. Libraries with a 1% PhiX spike were sequenced using a NextSeq 500/550 Mid Output Kit v2.5 300 cycle kit (Illumina, San Diego, CA, USA; #20024905), with a read length of 2 × 149 bp to provide a target of 1 to 5 million reads per library. The resulting sequence reads were analyzed via a custom bioinformatics pipeline as previously described [7]. 

Briefly, low quality bases were trimmed using Trimmomatic v0.38, and were then aligned to the NC_045512.2 reference genome using bwa mem v 0.7.17-r1188 [7]. Single nucleotide variants (SNVs) relative to the reference were detected using bcftools mpileup. SNVs occurring in at least 5% of the reads with at least five separate supporting instances were marked for further interrogation and SNVs specific to individual SARS-CoV-2 variants were assessed to determine their relative prevalence. Variant prevalence was calculated by assessing the relative percentages of individual SNPs associated with SARS-CoV-2 VOI/Cs; and the proportions of each specific VOI/C within a given sample was determined via the collective analysis of known SNP signatures or markers for each individual variant relative to each other.

### 2.4. Accession and Analysis of City of Louisville KY Public Health Data

Publicly available public health data was obtained via the accession of databases maintained by the Department of Public Health and Wellness (https://COVID-19-in-jefferson-county-ky-lojic.hub.arcgis.com/ (accessed on 16 September 2022)). All public health data used in this study may be found as part of the Table S1 accompanying this manuscript. Tabular data obtained from the Department of Health and Wellness was then limited to the time period relevant to this study and individual data entries were geocoded relative to their respective wastewater sheds and wastewater quality treatment centers (WQTCs; a term preferred for use by the Louisville/Jefferson County Metropolitan Sewer District, which is synonymous to wastewater treatment facility). The quantitative data for vaccinations administered, SARS-CoV-2 tests conducted, the number of positive tests reported to the Department of Public Health and Wellness, and deaths attributed to SARS-CoV-2 infection were summed on a weekly basis and divided by 7 to obtain the mean daily rate for each metric. As the individual WQTCs differ on the basis of population (as described in Table S1), the mean daily rates were divided by the population to determine the mean daily rates per 100 k population.

### 2.5. Statistical Analysis of the Impact of Delta Reemergence on SARS-CoV-2 Associated Deaths

To examine the correlation between the Delta reemergence events and SARS-CoV-2 associated deaths in the affected communities a three-week rolling average of SARS-CoV-2 associated deaths was applied to the data of each WQTC. Statistical assessment of the specific impact of the Delta reemergence on SARS-CoV-2 associated deaths was conducted by comparing the SARS-CoV-2 associated death rates for specific week periods where the prevalence of the Delta variant exceeded 20%; specifically, weeks 10, 12, 13, 15, and 18 met this criterion threshold. Statistical significance was determined by one-tailed Mann–Whitney tests of the unaffected WQTCs with the CCWQTC. To complement these data, similar analyses of data associated with week periods lacking the Delta variant were also tested. 

## 3. Results

### 3.1. The Power and Importance of Wastewater Monitoring in the Detection of Variant Emergence

**Figure 1:**
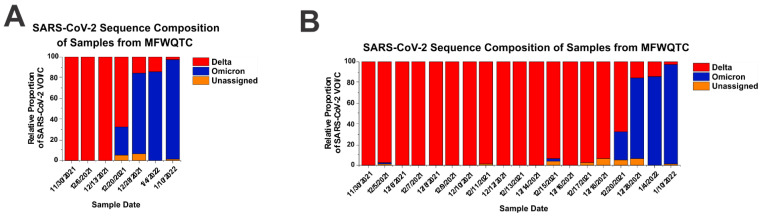
Weekly Monitoring of Aggregate Wastewater Samples is Sufficient to Promptly Detect SARS-CoV-2 Variant of Interest/Concern Emergence Events. (**A**) Samples of aggregate wastewater from the Morris Forman Wastewater Quality Treatment Center were collected as per the normal weekly sampling rate at the indicated dates and processed as described in the Materials and Methods. Shown is the relative proportions of individual SARS-CoV-2 VOI/Cs that exceeded a threshold of 1%. (**B**) As above in panel A; however, the samples were collected using a once-daily sampling schedule for a period of two weeks starting on 5 December 2021. Abutting this two-week daily sampling period is data obtained via the weekly sampling schedule, to add longer term context consistent with panel A. Dates shown on the above graphs follow a MM/DD/YYYY format.

Previously, we and others have established the importance of complementing clinical patient sampling along with geographical wastewater sampling for SARS-CoV-2 variants [7,13,14,15,16,17]. As reported in Rouchka and Chariker et al., 2021, surveillance efforts may be targeted to evaluate centralized wastewater quality treatment centers (WQTCs) to provide in depth genetic assessments in a quantitative fashion [7]. Such efforts allow for a collaborative approach to public health and a comprehensive appraisal of the current pandemic burden in the city of Louisville, KY and Jefferson County, KY at large [6]. Genomic sequencing in the community wastewater catchments permits a rapid, cost-effective way to identify circulating variants in an unbiased manner, as well the detection of any mutations of those variants before they are identified in clinical samples. Importantly, a major benefit of next-generation sequencing surveillance of SARS-CoV-2 over quantitative PCR methodologies is that next-generation sequencing and bioinformatic assessments do not require prior target identification, primer/probe generation and validation, and sample reassessment. Thus, as new variant strains of interest or concern emerge, the detection of their genetic signatures can be immediately added to the existing framework/analyses by including the detection of their unique or shared signature polymorphisms. As shown in Figure 1A, the retrospective and prospective adaptability of this approach enabled the detection of the emergence of the Omicron strain in Louisville KY. 

As the emergence of Omicron in the city of Louisville KY was anticipated during this time, the sampling frequency was increased from weekly to daily for a period of two-weeks to determine whether sampling frequency enhanced the capacity to detect the emergence of variants of concern in the community. As shown in Figure 1B, enhancing the frequency of sampling did not substantially enhance the capacity to detect early emergence of the Omicron variant in the community, as the first detection of Omicron signatures preceded the regular weekly sample capture by 4 days. Thus, increasing sampling frequency does not improve detection relative to the added cost.

### 3.2. Unexplained Periodic Reemergence of the Delta Strain in Wastewatersheds of Louisville KY

**Figure 2:**
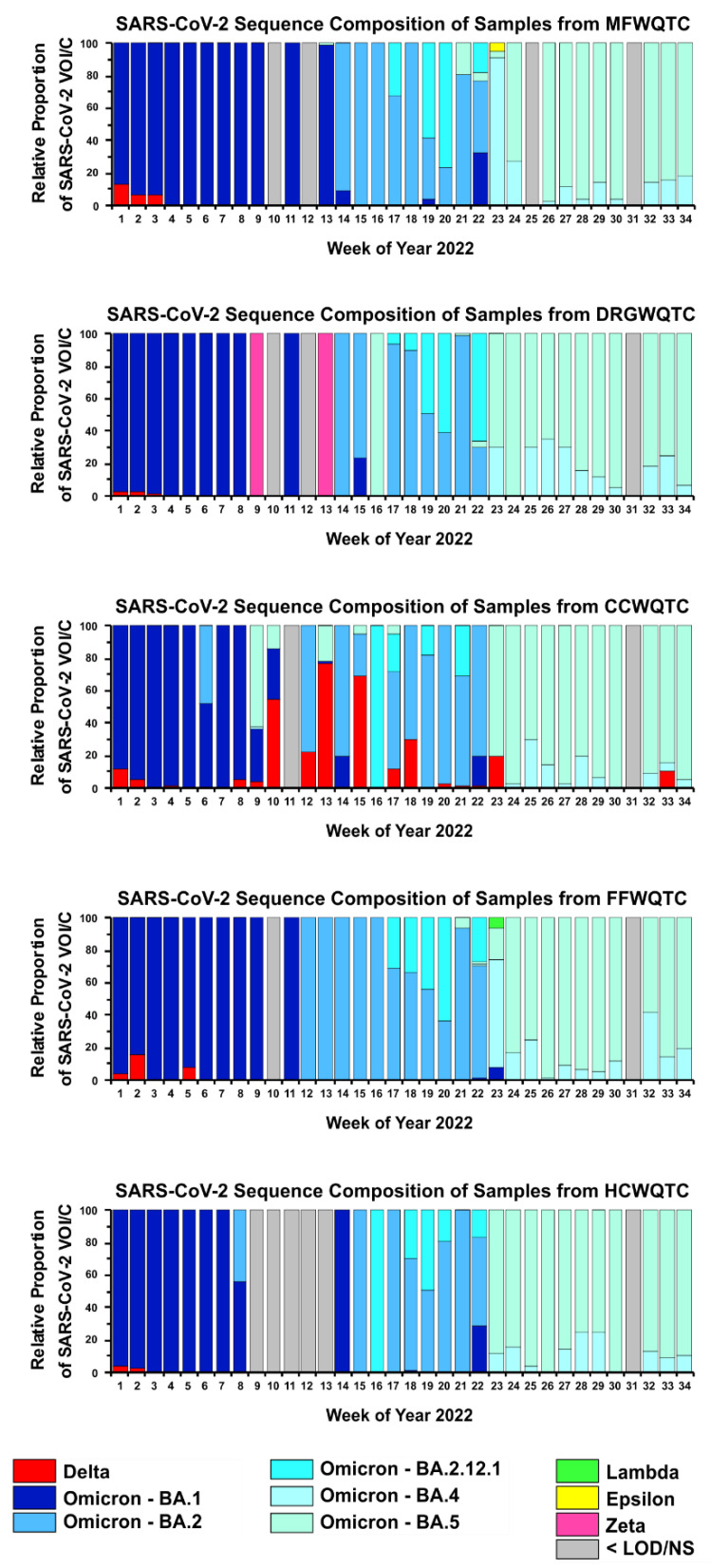
The Detection of Periodic Reemergence of the Delta Strain in Wastewatersheds with Next-Generation Sequencing. Samples of aggregate wastewater from each of the WQTCs serving the Louisville, KY population were collected and assessed for SARS-CoV-2 genetic composition on a weekly basis. The individual WQTCs include Morris Forman (MFWQTC), Derek R. Guthrie (DRGWQTC), Cedar Creek (CCWQTC), Floyd’s Fork (FFWQTC), and Hite Creek (HCWQTC). The specific week of 2022 pertaining to each sample is listed on the *X*-axis (with weeks defined as starting on Monday). Individual SARS-CoV-2 VOI/C are indicated by color-coding as indicated by the key at the bottom of the figure. Samples where the detection of SARS-CoV-2 was impaired due to low abundance, sample quality, or a lack of access to a sample are identified as <LOD/NS. Data has been limited to show the relative proportions of individual SARS-CoV-2 VOI/Cs that exceeded a threshold of 1% at any point, in any WQTC, during the indicated time period.

Genomic wastewater analysis of samples obtained from the five wastewater quality treatment centers indicated that after the dominant emergence of the Omicron variant in January of 2022, reemergence events of the Delta variant were detected three times, corresponding to the third week of February 2022 (week 8 of 2022); the first week of June 2022 (week 23 of 2022); and the third week of August 2022 (week 33 of 2022), in samples obtained from a single aggregate wastewater quality treatment center. Specifically, as shown in Figure 2, wastewater genomics monitoring for the period starting at the onset of year 2022 indicated a trickling reemergence of the Delta strain in wastewater of the Cedar Creek WQTC (CCWQTC) in the third week of February 2022 (week 8 of 2022). The reemergence of Delta impacted a significant number of individuals in the CCWQTC sewershed, as the Delta variant accounted for >50% of the polymorphism signatures in wastewater samples obtained from the CCWQTC catchment area for several weeks during the reemergence event. During this time the Omicron strain (including Omicron sublineages) dominated all other WQTCs, as was common for many other communities (including those specific to the Louisville metropolitan area and Jefferson County KY as a whole). Curiously, the February reemergence of the Delta strain in the CCWQTC persisted in the community persisted for 10 weeks until the first week of May 2022 (week 18 of 2022), when Omicron sublineages BA.2 and BA.2.12.1 were dominate in the community. Since this initial reemergence event, the Delta variant has reemerged twice again in the wastewater samples of the CCWQTC. Unlike the initial reemergence of the Delta variant, these events, which centered around the first week of June 2022 (week 23 of 2022), and the thirs week of August 2022 (week 33 of 2022), did not persist in the community as all polymorphism signatures quickly dissipated to give way to the Omicron BA.4 and BA.5 subvariants in the weeks immediately following the reemergence events. 

As mentioned earlier, the monitoring of SARS-CoV-2 prevalence and genetic variation in the wastewater of Louisville KY has been instrumental towards a complete understanding of the pandemic in the community [5,7]. A long-term goal of the partnership between the academic and public health missions has included understanding the seeding and spreading of variants in the community and discerning the role of vaccinations in the ability to stop the spread of certain variants. Within this framework the reemergence of a non-dominant strain in the CCWQTC sewershed during the summer period raised an alarm amongst the partners involved in the monitoring of the Louisville wastewater for public health.

**Figure 3:**
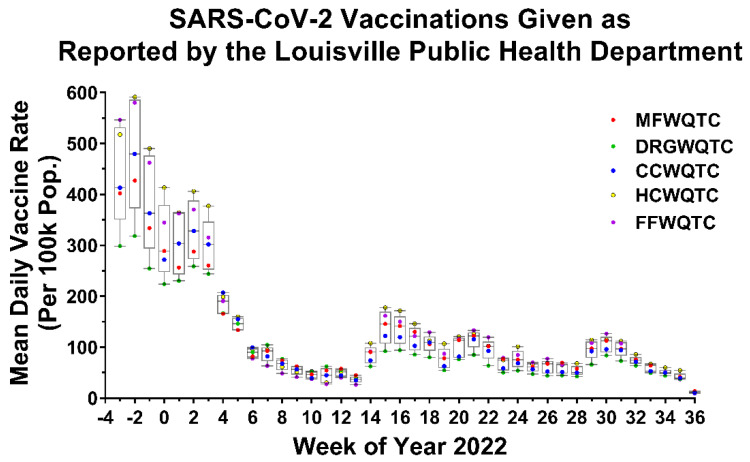
Daily Vaccination Rate of Communities Specific to the Individual Wastewater Quality Treatment Centers. Public health data was assessed to determine the number of vaccination events within each community served by the indicated WQTCs for a period preceding the initial Delta reemergence event by approximately six months (numbered relative to year 2022). The Tukey’s Box and whisker plots are inclusive of all communities, and individual data points for each WQTC are indicated. For Tukey Box and Whisker Plots the error bars denote the minimum and maximum values, and the boxed area represents the Interquartile Range (from the 1st to the 3rd quartiles), and the line represents the Median of the data set.

As the vaccines currently available at the time were known to be effective in limiting the health impacts of the Delta variant, we first examined whether vaccine uptake in the communities of Louisville, KY correlated with the reemergence events of the Delta variant. As shown in Figure 3, the communities associated with the CCWQTC consistently had lower relative vaccination rates than other regions; however, lower vaccine uptake was observed in other communities not experiencing Delta reemergence events (such as those serviced by the Derek R. Guthrie WQTC). Thus, the reemergence events of Delta are not necessarily due to low community vaccination rates. 

**Figure 4:**
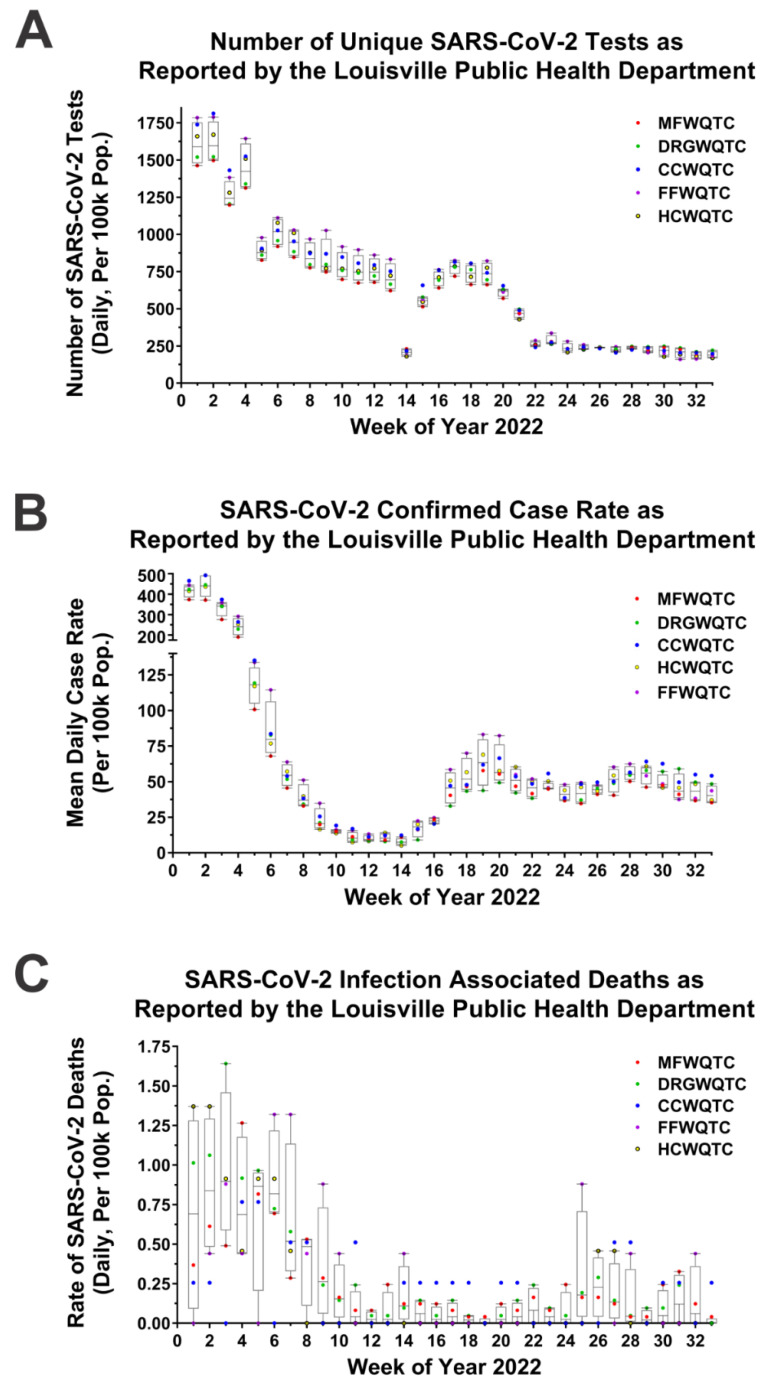
Retrospective Analysis of Public Health Data Reveals the Ongoing Impact of the Delta Variant to Public Health. Public health data was assessed to determine the mean daily number of SARS-CoV-2 tests, the mean daily number of positive SARS-CoV-2 tests, and the mean daily number of SARS-CoV-2 attributed deaths, per 100 k population for each of communities served by the indicated WQTCs (Panels (**A**–**C**)). To enable the rapid comparison of affected and unaffected communities, the Tukey’s Box and whisker plots are derived from the data pertaining to the four WQTCs not exhibiting Delta reemergence as reemergence may impact the above metrics, and individual data points for each WQTC are indicated. For Tukey Box and Whisker Plots the error bars denote the minimum and maximum values, and the boxed area represents the Interquartile Range (from the 1st to the 3rd quartiles), and the line represents the Median.

To assess whether the reemergence of the Delta strain negatively impacted community health the testing and clinical incidence of SARS-CoV-2 infections with respect to geographic location and time was examined. As shown in Figure 4A, the number of tests conducted across the WQTC regions were more or less equivalent throughout the individual weeks in question; however, the testing rates associated with the CCWQTC occasionally ranked amongst the highest during the periods when the Delta variant was present. It should be noted that these data do not include at-home tests, which were readily available at this time. Test positivity rates exhibited similar trends, with increases in relative positivity rate for the CCWQTC during times when the Delta variant was present. Examinations of clinical cases reported to the Louisville Public Health Department, as per Figure 4B,C, indicate that the CCWQTC did occasionally exhibit increased clinical incidence during the periods when the Delta strain was in reemergence. Notably, at weeks 5, 10, 11, 14, 23, 29 and 30, the burden of SARS-CoV-2 cases were in excess of all other WQTCs lacking the reemergence of Delta. Collectively, these data infer that the Delta variant did specifically contribute to the public health burden of SARS-CoV-2 during these reemergence events.

**Figure 5:**
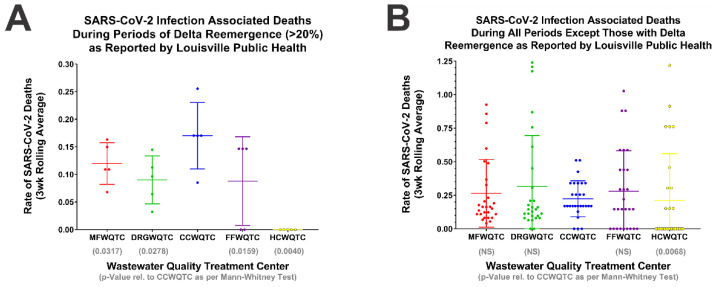
SARS-CoV-2 Reemergence Events Correlate with Increased Mortality in the Afflicted Wastewatershed Community. The public health data presented in Figure 4 was reassessed using a 3-week rolling average to determine the potential relationship between SARS-CoV-2 Delta reemergence and death. (**A**) Comparative analysis of the weeks with detected Delta reemergence in excess of 20%, and (**B**) comparative of all weeks exclusive of those associated with Delta reemergence. Individual data points represent individual weeks, with the lines indicating the mean and the error bars the standard deviations of the underlying data. Statistical analysis, as per the *p*-Values deriving from one-tailed Mann–Whitney tests, are indicated below the individual WQTC labels, and in respect to comparisons with the CCWQTC.

To better quantify and understand the impact of the Delta reemergence events on human health we examined the rate of SARS-CoV-2 associated deaths for the time periods in which the Delta variant accounted for >20% of wastewater prevalence. As SARS-CoV-2 deaths typically occur several weeks after the initial diagnosis, a three-week rolling average was applied to determine potential adverse outcomes associated with Delta reemergence events [18]. As shown in Figure 5A, during the weeks following Delta reemergence, SARS-CoV-2 associated deaths were increased in the CCWQTC community relative to all other WQTCs. To determine whether this difference was due to unknown confounding factors associated with the individual wastewatershed communities (as in to identify whether individuals in the CCWQTC community were more likely to succumb to SARS-CoV-2 infection), we similarly assessed all time periods unassociated with the Delta reemergence events. As depicted in Figure 5B, individuals in the CCWQTC wastewatershed were slightly less likely to experience adverse outcomes of SARS-CoV-2 infection; however, these trends lack any statistical power. 

## 4. Discussion

### 4.1. Weekly Monitoring of Wastewater Is Sufficient and Cost-Effective

At this stage of the SARS-CoV-2 pandemic there have been renewed discussions as to the importance of continuing pathogen surveillance efforts using aggregate or sewershed-specific wastewater monitoring. As demonstrated by the data presented here, the SARS-CoV-2 pandemic continues to be a dynamic situation in regard to the circulation of variants of concern/interest, despite the ongoing dominance of the Omicron lineage. Nonetheless, the cost–benefit comparisons for many communities play a significant role in public health decisions, as finite resources must be used to instill the greatest value to the communities which they serve. Previously we have reported that focusing efforts on sampling aggregate wastewater sources, such as wastewater quality treatment centers, provides a community level assessment of SARS-CoV-2 burden and genetic composition in an unbiased manner [7]. Here, we continued these assessments and sought to determine whether sampling frequency enhanced the capacity of wastewater monitoring to detect the emergence of variants of concern/interest in the community. As shown above, at least for Louisville KY, a weekly rate of sampling provided the best ratio of early detection and cost, as the increased resolution afforded by daily sampling did not appreciably improve detectability or indicate significant daily variations in SARS-CoV-2 genetic composition. Thus, for many communities, especially those with limited resources, weekly monitoring of an aggregate wastewater facility may provide the greatest insight into community SARS-CoV-2 burdens and dynamics. 

Our SARS-CoV-2 wastewater monitoring group has selected next-generation sequencing over the use of RT-qPCR methodologies to monitor VOI/C prevalence. While similar results may be obtained using RT-qPCR, the next-generation sequencing approach has several notable advantages. First, next-generation sequencing approaches have inherent flexibilities that RT-qPCR cannot provide, most importantly is the capacity to perform unbiased analyses without a need for prior target identification, probe generation, probe validation, and the determination of detection efficiency for each qPCR primer/probe set. As alluded to above, next-generation sequencing enables the detection of known and unknown polymorphism signatures without relying on the specific detection of individual polymorphisms through a targeted means. Thus, wastewater sequencing is superior to RT-qPCR as it is a readily deployable platform that requires no forethought as to target detection. Secondly, next-generation sequencing based approaches build historical catalogs of SARS-CoV-2 wastewater variation data which can be reexamined at later dates to determine if, and when, specific polymorphisms were present. Detection approaches based on RT-qPCR platforms would require reassessment of the individual cDNAs (including the regeneration of new cDNA if supplies were insufficient) to provide such information. Moreover, communities relying on RT-qPCR would need to decide whether continuing with specific VOI/C primer/probe sets in the absence of known infections in the community was an effective use of resources. As the Omicron variant is highly dominant one could envision a scenario where RT-qPCR schemes to detect Delta were discontinued, which would preclude the detection of the events reported here in this manuscript. Third, multiplexing allows for the costs of next-generation sequencing to be defrayed over many samples, driving the per run costs down to the point where per reaction costs are competitive with those associated with RT-qPCR in the long term. Finally, next-generation sequencing enables the identification of novel variants in real time, whereas RT-qPCR detection paradigms do not allow for the unbiased detection of novel variants at any stage of the process. Altogether, it is for these reasons that we posit that the costs and benefits of next-generation sequencing are superior to those of RT-qPCR based approaches. 

A considerable benefit of the RT-qPCR method is that many communities may lack access to the necessary equipment and expertise for next-generation sequencing, and investments in RT-qPCR technologies require smaller capital investments. Nonetheless, many partners in global public health are actively seeking to remove the financial barriers to these efforts for smaller communities so that the benefits of next-generation sequencing are accessible. 

### 4.2. Low Vaccine Uptake Does Not Correlate with Reemergence of the SARS-CoV-2 Delta Strain, and Reemergence Correlates with Increased Negative Outcomes to Public Health

As demonstrated by the data above, notable reemergence events involving the Delta strain of SARS-CoV-2 were observed in Louisville, KY after the introduction of Omicron variants into the community. However, it is important to note that efforts to sequence individual clinical specimens in the state of Kentucky have been limited in scope, and thus a complementary set of clinical isolate sequences from the period and location in question does not exist. Hence, we are not able to directly confirm the presence of clinical Delta infections during these reemergence events. While signatures of the Delta variant were readily detected in the community, the overall impacts of Delta reemergence were muted relative to historical expectations deriving from the first Delta wave. While public health data from the afflicted wastewatershed did reflect increases in clinical incidence and mortality during the reemergence events, the overall magnitudes of negative health outcomes were disproportionate suggesting that infections associated with Delta reemergence events are milder than those of the initial Delta wave [19]. The cause of milder Delta-associated disease is unclear at this time, but likely stems at least in part from vaccination, enhanced direct and indirect supportive care, prior infections with Omicron, or the ongoing circulation of Omicron in the community [12]. Although it should be noted that it is unknown as to whether those in the CCWQTC infected with the Delta variant had been infected prior with other variants, including Omicron, or if these individuals were genuinely immunologically naïve cases of infection.

Surprisingly, these reemergence events have not been uniformly observed across the Louisville metropolitan area, despite a clear history of interregional spread of SARS-CoV-2 variants in the Louisville communities. This observation is puzzling for several reasons, one of which is that areas with lower relative vaccination rates were not affected by the reemergence of the Delta variant. The precise reason behind this phenomenon is unknown, as the CCWQTC and DRGWQTC (which has the lowest relative vaccination rate of Louisville KY) are neighboring regions that have largely similar demographics, median household incomes, and population densities. Specifically, the CCWQTC serves a community of roughly 56,000 individuals with an estimated number of individual households of 21,500, as per the most current US Census data. These demographic values are in line with those of FFWQTC and HCWQTC with these wastewatershed communities being slightly smaller in comparison to CCWQTC as they serve populations of approximately 32,500 and 31,200, respectively. In contrast, the MFWQTC and DRGWQTC serve much larger and comparatively more diverse populations, with populations of approximately 350,000 and 296,000, respectively. Due to population size, it remains possible that a minority of cases may have a disproportionate effect within smaller wastewatersheds; however, as wastewater sequencing revealed the emergence of the Omicron variant within the MFWQTC community we have confidence that a failure to detect reemergence of the Delta variant in other wastewatersheds was not strictly a function of population size. Regardless, despite the underlying causes being unknown, reemergence of Delta events in the background of Omicron are clearly possible, and capable of negatively impacting community health [12,20]. Cryptic circulation of Delta variants in the presence of Omicron is not a phenomenon unique to the Louisville metropolitan area, as Delta polymorphism signatures have been detected in populations where the dominant variant was transitioning from Delta to Omicron using RT-qPCR methodologies [21,22]. Nonetheless, the reemergence events reported here, to the best of our knowledge, are unique in that they occur well after the change to Omicron variant dominance in the community. Whether these reemergence events are due to the emergence or presence of Deltacron variants, where the SNPs associated with Delta and Omicron appear in the same viral genome, is of particular interest. Unfortunately, due to the fragmented nature of amplicons generated by wastewater sequencing efforts it is impossible to state with certainty whether this is contributing to the phenomena observed here as definitive evidence would require the identification of all SNP signatures in a single RNA molecule. However, the SNP signatures reported here do not correlate with each other suggesting that they are out of phase with one another and arise from separate genomes in the wastewater samples, as correlations between the relative prevalence would be anticipated if they arose from a single genome. As such, it is unlikely that these reemergence events are associated with cryptic variants. 

Whether reemergence events become more commonplace as vaccine and infection-associated immunity wanes with respect to time remains to be seen; however, the likelihood of such is high.

### 4.3. The Detection of SARS-CoV-2 Reemergence Events Underscores the Importance of Personalized Medicine in Infectious Disease

The data presented above indicates that despite the circulation of dominant SARS-CoV-2 strains, prior VOI/Cs may reemerge within a population with demonstrable negative consequences to individual health. As the SARS-CoV-2 standards of care are largely determined on a regional basis, reemergence events may constitute lost opportunities for clinical interventions if the standard lacks consideration of real time variant analysis of wastewater or pathogen sequence analyses in the individual patient. For instance, the shift towards Omicron dominance changed the standard of care to utilize therapeutic monoclonal antibodies with known specific impacts to the Omicron variant [23]. Thus, individuals with SARS-CoV-2 Delta infections would be at a disadvantage clinically, as they may receiving therapies which are less effective against their specific pathogen. Whether or not tailored interventions would have altered the outcomes of the Delta reemergence events described here is impossible to know; however, the recognition of significant reemergence events in the community exposes the importance of understanding the specificities of pathogen variation in the finite communities, and more importantly the individual, in regard to infectious disease. 

### 4.4. Forward Looking to the Next Pandemic

While the burden posed by SARS-CoV-2 remains ongoing and significant, contemporary outbreaks of Monkeypox and Poliovirus have renewed interest in wastewater pathogen surveillance [24,25,26]. As demonstrated by our work, and that of many others, the robust and unbiased nature of wastewater surveillance will undoubtedly be a strong tool with which future outbreaks may be identified, and public health efforts focused. Therefore, partnerships between academic, governmental, and nonprofit organizations are vital to the continuing development of innovative strategies with which communities may prepare for the next pandemic, regardless as to whether it be viral or bacterial in nature. 

## 5. Conclusions

As the COVID-19 pandemic is now globally reaching an endemic status, it is important to continue efforts to characterize infection rates and pathogen genetic variation within communities by wastewater surveillance efforts. Continued monitoring has led to a comprehensive view of pandemic dynamics at a community level and has enabled the deployment of targeted public health interventions to increase public wellbeing. Additionally, a fine resolution of infection over a community level has allowed us to draw conclusions as to how vaccination status may allow or lead to the reemergence and continuation of variants of concern in a community. 

## Data Availability

Raw sequence data is available in NCBI’s Sequence Read Archive under BioProject number PRJNA735936.

## Supplementary Materials

The following supporting information can be downloaded at: https://www.mdpi.com/article/10.3390/pathogens11111249/s1, Table S1: Public Health Information Reported for the City of Louisville (Current as of 9/18/22) and related data.

## References

[1] Fontenele R.S., Kraberger S., Hadfield J., Driver E.M., Bowes D., Holland L.A., Faleye T.O.C., Adhikari S., Kumar R., Inchausti R. (2021). High-throughput sequencing of SARS-CoV-2 in wastewater provides insights into circulating variants. Water Res..

[2] Pecson B.M., Darby E., Haas C.N., Amha Y.M., Bartolo M., Danielson R., Dearborn Y., Di Giovanni G., Ferguson C., Fevig S. (2021). Consortium SA-C-I. Reproducibility and sensitivity of 36 methods to quantify the SARS-CoV-2 genetic signal in raw wastewater: Findings from an interlaboratory methods evaluation in the U.S. Environ.Sci. Water Res. Technol..

[3] Wu F., Zhang J., Xiao A., Gu X., Lee W.L., Armas F., Kauffman K., Hanage W., Matus M., Ghaeli N. (2020). SARS-CoV-2 Titers in Wastewater Are Higher than Expected from Clinically Confirmed Cases. Msystems.

[4] Holm R.H., Mukherjee A., Rai J.P., Yeager R.A., Talley D., Rai S.N., Bhatnagar A., Smith T. (2022). SARS-CoV-2 RNA abundance in wastewater as a function of distinct urban sewershed size. Environ.Sci. Water Res. Technol..

[5] Smith T., Holm R.H., Keith R.J., Amraotkar A.R., Alvarado C.R., Banecki K., Choi B., Santisteban I.C., Bushau-Sprinkle A.M., Kitterman K.T. (2022). Quantifying the relationship between sub-population wastewater samples and community-wide SARS-CoV-2 seroprevalence. Sci. Total Environ..

[6] Smith T., Holm R.H., Yeager R., Moore JBt Rouchka E.C., Sokoloski K.J., Elliott E.M., Talley D., Arora V., Moyer S., Bhatnagar A. (2022). Combining Community Wastewater Genomic Surveillance with State Clinical Surveillance: A Framework for SARS-CoV-2 Public Health Practice. Food Environ. Virol..

[7] Rouchka E.C., Chariker J.H., Saurabh K., Waigel S., Zacharias W., Zhang M., Talley D., Santisteban I., Puccio M., Moyer S. (2021). The Rapid Assessment of Aggregated Wastewater Samples for Genomic Surveillance of SARS-CoV-2 on a City-Wide Scale. Pathogens.

[8] Dhawan M., Saied A.A., Mitra S., Alhumaydhi F.A., Emran T.B., Wilairatana P. (2022). Omicron variant (B.1.1.529) and its sublineages: What do we know so far amid the emergence of recombinant variants of SARS-CoV-2?. Biomed. Pharm..

[9] Lyke K.E., Atmar R.L., Islas C.D., Posavad C.M., Szydlo D., Paul Chourdhury R., Deming M.E., Eaton A., Jackson L.A., Branche A.R. (2022). Rapid decline in vaccine-boosted neutralizing antibodies against SARS-CoV-2 Omicron variant. Cell Rep. Med..

[10] Anichini G., Terrosi C., Gori Savellini G., Gandolfo C., Barbagli F., Carta G.A., Fabrizi S., Miceli G.B., Cusi M.G. (2022). Antibody Response against Circulating Omicron Variants 8 Months after the Third Dose of mRNA Vaccine. Vaccines.

[11] Amati R., Frei A., Kaufmann M., Sabatini S., Pellaton C., Fehr J., Albanese E., Puhan M.A. (2022). Functional immunity against SARS-CoV-2 in the general population after a booster campaign and the Delta and Omicron waves, Switzerland, March 2022. Eurosurveillance.

[12] Khan K., Karim F., Cele S., Reedoy K., San J.E., Lustig G., Tegally H., Rosenberg Y., Bernstein M., Jule Z. (2022). Omicron infection enhances Delta antibody immunity in vaccinated persons. Nature.

[13] La Rosa G., Mancini P., Bonanno Ferraro G., Veneri C., Iaconelli M., Lucentini L., Bonadonna L., Brusaferro S., Brandtner D., Fasanella A. (2021). Rapid screening for SARS-CoV-2 variants of concern in clinical and environmental samples using nested RT-PCR assays targeting key mutations of the spike protein. Water Res..

[14] Bi C., Ramos-Mandujano G., Tian Y., Hala S., Xu J., Mfarrej S., Esteban C.R., Delicado E.N., Alofi F.S., Khogeer A. (2021). Simultaneous detection and mutation surveillance of SARS-CoV-2 and multiple respiratory viruses by rapid field-deployable sequencing. Med.

[15] Crits-Christoph A., Kantor R.S., Olm M.R., Whitney O.N., Al-Shayeb B., Lou Y.C., Flamholz A., Kennedy L.C., Greenwald H., Hinkle A. (2021). Genome sequencing of sewage detects regionally prevalent SARS-CoV-2 variants. MBio.

[16] Martin J., Klapsa D., Wilton T., Zambon M., Bentley E., Bujaki E., Fritzsche M., Mate R., Majumdar M. (2020). Tracking SARS-CoV-2 in sewage: Evidence of changes in virus variant predominance during COVID-19 pandemic. Viruses.

[17] Nemudryi A., Nemudraia A., Wiegand T., Surya K., Buyukyoruk M., Cicha C., Vanderwood K.K., Wilkinson R., Wiedenheft B. (2020). Temporal detection and phylogenetic assessment of SARS-CoV-2 in municipal wastewater. Cell Rep. Med..

[18] Challen R., Brooks-Pollock E., Read J.M., Dyson L., Tsaneva-Atanasova K., Danon L. (2021). Risk of mortality in patients infected with SARS-CoV-2 variant of concern 202012/1: Matched cohort study. BMJ.

[19] Sigal A., Milo R., Jassat W. (2022). Estimating disease severity of Omicron and Delta SARS-CoV-2 infections. Nat. Rev. Immunol..

[20] Yaniv K., Ozer E., Shagan M., Paitan Y., Granek R., Kushmaro A. (2022). Managing an evolving pandemic: Cryptic circulation of the Delta variant during the Omicron rise. Sci. Total Environ..

[21] Wolfe M., Hughes B., Duong D., Chan-Herur V., Wigginton K.R., White B.J., Boehm A.B. (2022). Detection of SARS-CoV-2 Variants Mu, Beta, Gamma, Lambda, Delta, Alpha, and Omicron in Wastewater Settled Solids Using Mutation-Specific Assays Is Associated with Regional Detection of Variants in Clinical Samples. Appl. Environ. Microbiol..

[22] Lee W.L., Armas F., Guarneri F., Gu X., Formenti N., Wu F., Chandra F., Parisio G., Chen H., Xiao A. (2022). Rapid displacement of SARS-CoV-2 variant Delta by Omicron revealed by allele-specific PCR in wastewater. Water Res..

[23] Takashita E., Kinoshita N., Yamayoshi S., Sakai-Tagawa Y., Fujisaki S., Ito M., Iwatsuki-Horimoto K., Chiba S., Halfmann P., Nagai H. (2022). Efficacy of Antibodies and Antiviral Drugs against Covid-19 Omicron Variant. N. Engl. J. Med..

[24] de Jonge E.F., Peterse C.M., Koelewijn J.M., van der Drift A.R., van der Beek R., Nagelkerke E., Lodder W.J. (2022). The detection of monkeypox virus DNA in wastewater samples in the Netherlands. Sci. Total Environ..

[25] Zuckerman N.S., Bar-Or I., Sofer D., Bucris E., Morad H., Shulman L.M., Levi N., Weiss L., Aguvaev I., Cohen Z. (2022). Emergence of genetically linked vaccine-originated poliovirus type 2 in the absence of oral polio vaccine, Jerusalem, April to July 2022. Eurosurveillance.

[26] Link-Gelles R., Lutterloh E., Schnabel Ruppert P., Backenson P.B., St George K., Rosenberg E.S., Anderson B.J., Fuschino M., Popowich M., Punjabi C. (2022). Public Health Response to a Case of Paralytic Poliomyelitis in an Unvaccinated Person and Detection of Poliovirus in Wastewater—New York, June–August 2022. MMWR.

